# Driving performance and ocular activity following acute administration of 10 mg methylphenidate: A randomised, double-blind, placebo-controlled study

**DOI:** 10.1177/02698811241286715

**Published:** 2024-10-11

**Authors:** Blair Aitken, Luke A Downey, Serah Rose, Thomas R Arkell, Brook Shiferaw, Amie C Hayley

**Affiliations:** 1 Centre for Mental Health and Brain Sciences, Swinburne University of Technology, Hawthorn, VIC, Australia; 2 Institute for Breathing and Sleep, Austin Health, Heidelberg, VIC, Australia; 3 Seeing Machines, Fyshwick, ACT, Australia

**Keywords:** Methylphenidate, ocular monitoring, driving performance, road safety, psychostimulants

## Abstract

**Background:**

Methylphenidate is a routinely prescribed treatment for attention-deficit/hyperactivity disorder with misuse potential owing to its perceived performance-enhancing and euphoric properties. Although clinically effective, there is limited understanding of how methylphenidate affects safety-sensitive tasks such as driving when used by healthy individuals.

**Aim:**

Explore the acute effects of 10 mg methylphenidate on driving performance and gaze behaviour.

**Methods:**

Twenty-five fully licensed, healthy adults (mean age = 33.5 ± 7.8 years, 64% male) took part in two 40-min simulated highway drives with simultaneous eye movements monitored using a proprietary automotive-grade driver monitoring system (Seeing Machines). Driving performance was assessed using the standard deviation of lateral position, standard deviation of speed and steering variability. Visual scanning efficiency was determined using ocular metrics, such as fixation duration and rate, gaze transition entropy, and stationary gaze entropy, were assessed to determine visual scanning efficiency.

**Results:**

Methylphenidate significantly improved driving performance by reducing lane weaving and speed variation, particularly in the latter half of the drive. Although a significant reduction in fixation duration was observed, all other ocular metrics remained unchanged.

**Conclusions:**

Methylphenidate mitigates performance decrements typically associated with prolonged and monotonous driving. The absence of pronounced oculomotor effects suggests that a single 10 mg dose of methylphenidate has no deleterious impact on visual scanning behaviour during driving tasks with low-to-moderate cognitive demand. Future research should investigate the effects of methylphenidate under various dosing and driving conditions to better understand its impact.

**Trial Registration:**

ACTRN12620000499987.

## Introduction

The increasing diagnosis of attention-deficit/hyperactivity disorder (ADHD) has led to a rise in the prescription of amphetamine-derivative stimulants, such as Adderall and Vyvanse and methylphenidate (e.g. Ritalin and Concerta) as first-line therapy options (Board et al., 2020). From 2001 to 2015, there was a considerable increase in the use of ADHD medication among adults, with the most significant increases reported in Australia (up to 25.06%), followed by northern Europe (18.81%, 2001–2013), western Europe (17.01%, 2001–2014), and the United States (14.30%, 2001–2010). Up to 90% of ADHD medication users are administered methylphenidate, commercially sold under the brand name Ritalin^®^ (Raman et al., 2018). Methylphenidate primarily exerts its effects by inhibiting dopamine and norepinephrine reuptake in presynaptic neurons in the brain and increasing catecholamine availability, thereby improving concentration and attention (Faraone, 2018). While generally effective when used as prescribed, 5 million adults in the US and 400,000 in Australia (both equating to 2.1% of adults) misuse prescription stimulants each year, including use without a prescription, in greater amounts, or for longer durations than prescribed (Australian Institute of Health and Welfare, 2024; Compton et al., 2018). This misuse is typically driven by a desire to experience off-target euphoric effects, prolonged wakefulness, or improved cognitive performance (Compton et al., 2018). This raises concerns about the broader implications of stimulant use, particularly in contexts of safety-sensitive tasks such as driving, where cognitive enhancement and risk-taking behaviours may intersect.

Every day, around 3250 people are killed in road traffic collisions (RTCs), with 43.6% of cases involving the use of a psychoactive substance (World Health Organisation, 2023, 2018). Over the last 2 decades, stimulant-impaired drivers have been consistently over-represented in road trauma statistics, with 10.6% of seriously wounded and 12.5% of fatally injured road users testing positive for either licit or illicit stimulants (Brady and Li, 2014; Su et al., 2022). While stimulants are broadly associated with an increase in risky driving (Hayley et al., 2019) and an elevated incidence of road trauma (Hayley et al., 2016), the specific impact of prescription psychostimulants, particularly methylphenidate, on driving performance is unclear. While previous research has shown that an acute dose of methylphenidate improved driving performance in ADHD patients after 3 days without treatment (Verster et al., 2008; Verster and Roth, 2013), the only study to directly investigate these effects in healthy adults found that a 20 mg dose improved vehicle control during a 60-min simulated highway task. Other important driving skills, such as speed maintenance and braking response time, were unaffected (Ramaekers et al., 2006). Given the prevalence of stimulant medication misuse among adults who drive regularly, there is a need to better understand how methylphenidate impacts driving abilities, particularly across different user populations and under various conditions of use, such as prescribed versus non-prescribed use and varying dosage levels.

Methylphenidate selectively targets neuroanatomical regions responsible for visuo-ocular behaviour, affecting visual attention and cognitive-perceptual processes involved in visual search (Hayley et al., 2021). Studies in healthy adults indicate that acute methylphenidate use enhances response time to targets in central vision but not to unexpected stimuli or those in peripheral vision (Allman et al., 2012). These findings lend support to the proposed ‘tunnel vision’ effect typically associated with psychostimulant use, characterised by narrowed gaze dispersion and restricted processing of the visual field (Hayley et al., 2021). In the context of driving, this phenomenon may lead to an increased risk of RTCs due to diminished responses to sudden or unexpected stimuli entering the visual field, such as a pedestrian stepping onto the street or cars approaching from side streets. Consequently, there is an urgent need for proactive interventions such as driver monitoring systems (DMSs). These systems, which utilise in-cabin cameras, observe the driver’s ocular activity, including blink patterns, head position, and pupil state, to detect impairment in real-time (Hayley et al., 2021).

Metrics such as stationary gaze entropy (SGE) and gaze transition entropy (GTE) are valuable in quantifying gaze predictability and distribution and have proven effective in detecting driving impairment due to alcohol (Shiferaw et al., 2019b) and benzodiazepine use (Aitken et al., 2023). By examining ocular metrics and driving performance together, the impact of methylphenidate’s distinct effect on visual functioning could offer a novel approach to identifying stimulant-related driving impairment in real time. Further, continuous monitoring of driving performance and ocular activity makes it possible to identify potential time-on-task effects that may arise and shed more light on the magnitude of the drug’s effect on driving performance and associated visuomotor skills. Such evidence may help disentangle the apparent discrepancy between the acute enhancements seen in neurocognitive performance and wakefulness with the disproportional representation of stimulant-positive drivers in RTCs causing injury or death.

This study investigates the acute effects of a 10 mg dose of methylphenidate on driving performance while simultaneously monitoring ocular activity in a simulated driving environment. Despite being a relatively low dose, it corresponds to typical introductory therapeutic levels, making this study relevant for elucidating the initial effects of methylphenidate on driving performance in individuals with minimal prior exposure to the drug. Specifically, the objectives were to (i) evaluate changes in driving performance outcomes, such as vehicle control and speed maintenance, following methylphenidate administration; (ii) assess alterations to gaze behaviour and fixation patterns as indicators of changes in the driver’s visuospatial engagement; and (iii) to explore the temporal dynamic of methylphenidate’s effects to determine whether these impacts on driving performance and ocular activity are consistent, fluctuate or diminish over time. Typically, fixation behaviour and optimality are assessed in relation to specific, competing targets; however, in the present study, there is no defined optimal fixation strategy, reflecting the more generalised nature of visual scanning during driving. By addressing these objectives, this research aims to enhance the current understanding of methylphenidate’s acute effects on driving performance and processes essential for driving such as visuospatial attention, thereby providing valuable insights for the development of road safety strategies.

## Methods

### Participants

A total of 25 healthy adults (16 males and 9 females, aged 23–47 years) were recruited from January 2022 to September 2023 through physical flyers, online advertisements and word of mouth. Eligibility criteria included being aged 21–45 years; possessing normal or corrected-to-normal vision; being a regular driver with more than 4000 km/year of driving experience and holding a full driver’s licence (excluding probationary or learner drivers); having a body mass index (BMI) between 18.5 and 30 kg/m^2^; and having a blood pressure below 160/100 mmHg. To avoid potential risks to their health, participants with a known past or present psychiatric illness, mood disorder, history of drug abuse or dependence or any other significant psychological condition were excluded. Participants were also screened for depression and anxiety using the Beck Depression Inventory (Beck et al., 1987) and Beck Anxiety Inventory (BAI) (Beck et al., 1993), with scores of ⩾20 and ⩾16, respectively, serving as exclusion criteria. Additionally, those currently taking psychoactive medication that might interact with the study treatment, or those who were pregnant, potentially pregnant or lactating, were excluded. All participants provided written informed consent before participating in the study.

### Design

In this randomised, double-blind, placebo-controlled crossover study, participants completed two experimental sessions, scheduled at least a week apart to minimise potential residual drug effects. Each participant received a 10 mg dose of methylphenidate (Ritalin^®^) (active) and a matching placebo capsule, with the sequence of treatments determined by pre-randomisation using the Research Randomiser Software Version 4.0 (Social Psychology Network, US). While we did not include a baseline driving condition preceding the administration of placebo or methylphenidate, the within-subjects design of the study mitigates some of this limitation by allowing each participant to serve as their own control. Researchers conducting the study were blinded to the study treatments and were neither involved in generating the randomisation schedule nor allocating participants to different drug conditions. The study was registered prospectively on the Australian New Zealand Clinical Trials Registry (ACTRN12620000499987) and was approved by Swinburne University’s Human Research Ethics Committee in accordance with the National Statement on Ethical Conduct in Human Research (2018; approval number 20202839-411).

### Apparatus

#### Driving simulator

The experiments were conducted on a SIMREX compact driving simulator (Innosimulation Co. Ltd., South Korea) coupled with UC-win/Road software (Forum8, Tokyo, Japan). This simulator features a stationary vehicle base with an adjustable seat, dashboard, steering wheel, indicators, gear level, brake and accelerator. Audio effects, including engine, braking and other driving sounds, are relayed through stereo speakers. The simulation was displayed directly in front of participants across three 65-inch monitors, with the driver’s seat positioned approximately 180 cm away. Driving data were sampled at a frequency of 20 Hz.

#### Driving scenario

The simulated environment replicated a 105-km, bi-directional, four-lane highway with standard Australian road markings and signage. Participants were required to drive for 40 min and maintain a steady position in the left lane at a constant speed of 100 km/h. Occasionally, they performed overtaking manoeuvres due to traffic conditions. This task has been shown to be sensitive to the effects of alcohol (Garrisson et al., 2022), benzodiazepines (Aitken et al., 2023) and medical cannabis (Manning et al., 2023, 2024).

#### Eye tracking

Drivers’ ocular events were recorded using Seeing Machine’s proprietary automotive-grade DMS at a sampling rate of 46 Hz. The driver-facing camera was mounted on the dashboard of the driving simulator, and the participant did not interact with the system at all. This system has previously been utilised in research to index gaze behaviour during driving (Cai et al., 2021; Radhakrishnan et al., 2023).

### Procedure

**Figure 1.**
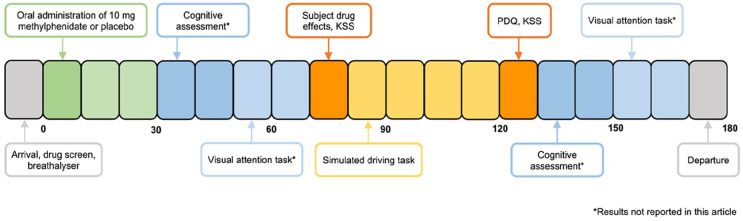
Overview of testing day procedures. Each block represents a 10-min interval. *Tasks whose results are not reported in this article.

Before enrolling, participants underwent an initial medical screening at Swinburne University of Technology. This included a 15-min explanatory interview, where a researcher outlined the study’s risks and requirements and obtained informed consent. This was followed by a medical history review and a physical examination with a clinical research nurse, which was later confirmed by a study physician. Participants then performed a 5-min practise driving task, before being scheduled for their two testing visits. They were instructed to fast for 2 h (water allowed), avoid caffeine for 12 h, and abstain from alcohol and nicotine for 24 h prior to each experimental session. Additionally, participants were instructed to avoid psychoactive drugs and contraindicated medication use throughout the study. Prior to treatment provision, all participants underwent a baseline breathalyser to confirm a zero-breath alcohol concentration and were screened for tetrahydrocannabinol, benzodiazepines, cocaine, amphetamines and opiates using a Securetec 6S DrugWipe Each participant received $50 reimbursement per session, a transportation voucher and information on potential side effects before being sent home. The duration of each testing visit was approximately 3 h (Figure 1).

### Driving performance

Driving performance was assessed at 85-min post-dosing. The primary outcome measure was the standard deviation of lateral position (SDLP), which is commonly regarded as the most reliable and sensitive metric for measuring driving performance following the administration of central nervous system drugs (Verster and Roth, 2011). It is calculated using the standard deviation of the distance (in cm) from the centre of the lane over the course of the drive, with higher values indicating greater deviations from the lane centre or ‘weaving’. To ensure accuracy, data points collected during overtaking manoeuvres were excluded to reduce the impact of intentional lane departures. Specifically, only data from the left lane during straight road segments were included in the analysis. Driving performance was also evaluated using the standard deviation of speed (SDS), measured in km/h and steering variability, which is defined as the standard deviation of the steering column angle with values ranging from zero to one.

### Ocular outcomes

The key ocular parameters assessed during the driving task were fixation duration, fixation rate (count per minute), SGE and GTE. SGE calculates the level of uncertainty in the spatial distribution of gaze by applying Shannon’s entropy computation (Shannon, 1948) to the probability distribution of fixation locations within the valid area of interest. Higher values indicate a wider distribution of fixations, implying a more dispersed gaze, while lower values indicate a more focused gaze. GTE computes an overall measure of predictability of visual scanning patterns by applying the conditional entropy equation to Markov chain matrices of fixation transitions. Higher values suggest a less structured, or more random, scanning pattern (Krejtz et al., 2014).

### Subjective measures

#### Drug effects

At 80-min post-dosing, participants rated drug intensity (‘strong’), feeling (‘good’ and ‘bad’) and appeal (‘like’) using separate five-point Likert-type scales. These scales ranged from ‘not at all’ to various degrees of ‘extremely’ (e.g. ‘very strong effect’ or ‘very much’). The Karolinska Sleepiness Scale (KSS) (Shahid et al., 2011) was used to assess psycho-physical state at 80- and 120-min post-dosing, with responses ranging from 1 (‘extremely alert’) to 9 (‘very sleepy, struggling immensely to remain awake, fighting sleep’).

#### Perceived driving quality and simulator sickness

Immediately following the driving task (at 120-min post-dosing), participants rated their driving performance using a Visual Analogue Scale ranging from 0 (‘I drove exceptionally poorly’) to 20 (‘I drove exceptionally well’) around a midpoint of 10 (‘I drove normally’). Additionally, to monitor potential motion-induced sickness resulting from the driving simulator, participants completed the Driving Simulator Sickness Questionnaire (DSSQ) (Kennedy et al., 1993). The DSSQ consists of 16 symptoms, such as fatigue, eye strain, headache and vertigo, each rated on a four-point Likert-type scale ranging from 0 (‘none’) to 3 (‘severe’).

### Data processing

Initially, fixations were identified by parsing raw ocular data in R (Core Team, 2021) with the *emov* package (created by Simon Schwab in 2016). This algorithm was configured to identify fixations exhibiting a maximum dispersion of 1° and a duration between 200 and 2000 ms. Fixations falling outside a predefined 100 by 50 px area of interest were also excluded. To calculate SGE and GTE, the valid area of interest was segmented into 280 equal state spaces, resulting in a maximum entropy of log_2_(280) = 2.79, which was used to normalise scores. This grid was designed to capture the critical visual area needed for a driver to maintain a steady lane position and speed while excluding non-pertinent values in GTE calculations. Entropy values (measured in bits) were normalised by dividing by the maximum possible entropy, with 0 being minimal entropy and 1 representing maximum entropy, as recommended by Shiferaw et al. (2018). Given the disparate sampling rates of the DMS and the driving simulator, data were aggregated into 10-min chunks to provide sufficient tolerance for these differences.

### Statistical analysis

Prior to conducting our analysis, we assessed the data for completeness and examined standardised residual values, specifically targeting outliers less than or greater than three standard deviations from the mean. This examination revealed no outliers for which exclusion from our analyses was warranted. Due to equipment failure, driving data for two participants were missing for one or more 10-min interval, and ocular data for six participants were incomplete for at least one study visit. This missing data, classified as missing completely at random, was not related to treatment effects or study outcomes. Consequently, we conducted our analyses with and without participants with incomplete data, which resulted in only minor differences in mean values and variances across outcomes. As such, we chose to retain participants with missing data in the final analyses.

Linear mixed models with the restricted maximum likelihood estimation were used to assess the effect of treatment (methylphenidate vs placebo), time (categorised into 10-min driving intervals) and their interaction on driving performance (SDLP, SDS and SV) and ocular outcomes (SGE, GTE, fixation duration and fixation rate). The optimal variance structure for the model was determined using likelihood ratio statistics, including the −2 log-likelihood and Akaike’s Information Criterion. Three variance structures were examined, including Autoregressive (1), Compound Symmetry and Diagonal. Compound symmetry was identified as the best fit as it exhibited the lowest Information Criteria values. Subjects were added to the model as a random effect, with both treatment and time entered as repeated measures variables. Separate models were built for each driving and ocular outcome. When a main effect was observed, Bonferroni-corrected pairwise comparisons were performed to contrast each treatment and time point. Paired-sample *t*-tests were performed to investigate differences in subjective measures (drug effects, KSS, PDQS and SSQ) between and within treatments. All analyses were conducted using IBM Statistical Package for Social Sciences 29.0 (SPSS Inc., USA) with statistical significance defined as *p* < 0.05.

## Results

**Figure 2.**
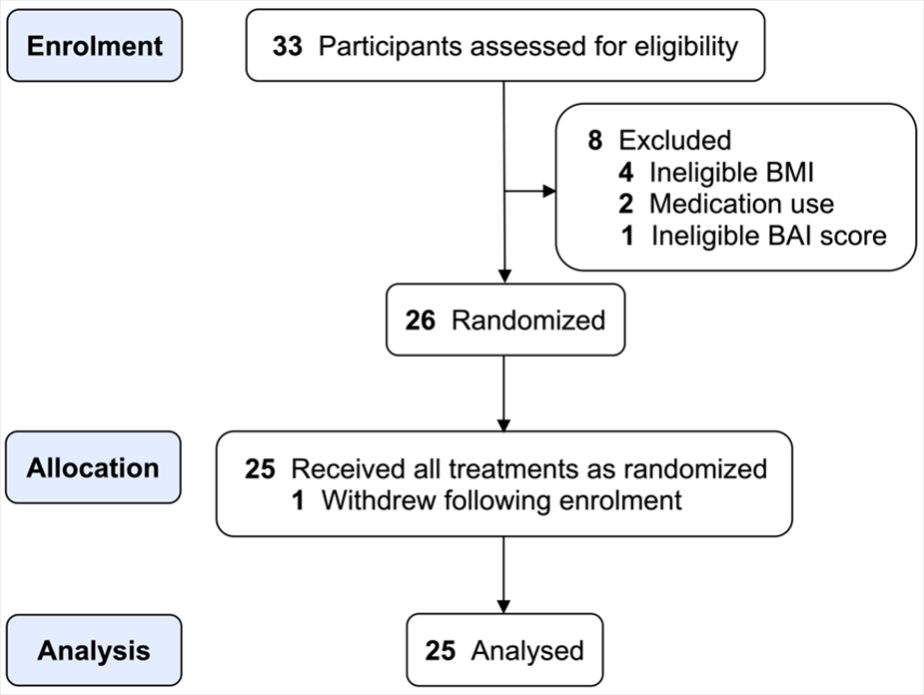
CONSORT diagram illustrating the flow of participant recruitment.

Figure 2 presents a Consolidated Standards of Reporting Trials diagram illustrating the participant recruitment flow. Of the 33 individuals screened for eligibility, four exceeded the BMI criterion, two were ineligible due to use of prescription medications not permitted in the study, one exceeded the BAI threshold and one withdrew after enrolling in the trial. The remaining 25 participants completed both the placebo and methylphenidate arms of the study with no attrition. Table 1 presents the characteristics of the 25 participants included in the analysis. The mean age of participants was 33.5 years (SD ± 7.8) with a BMI of 24.1 kg/m^2^ (SD ± 2.9).

**Table 1.** Baseline characteristics of the 25 participants.

Characteristics	*n* (%)	Mean (±SD) (range)
Gender		
Male	16 (64)	
Female	9 (36)	
Age (years)		33.5 (7.8) (23–47)
BMI (kg/m^2^)		24.1 (2.9) (18.6–31)
Ethnicity		
Caucasian	22 (88)	
Asian	3 (12)	
Participants with at least a tertiary education	23 (92)	
Drug use history*		
Amphetamines/speed	9 (36)	
MDMA/ecstasy	10 (40)	
Cocaine	11 (44)	
Alcohol	25 (100)	
Cannabis	22 (88)	
Inhalants	1 (4)	
Driving experience		
Years holding driver’s licence, years		10.5 (7.9) (1–27)
Weekly driving distance (km)		316.3 (232.6) (50–800)
Self-rated experienced driver	24 (96%)	

* Lifetime use only.

The majority of participants (88%) were Caucasian, with 92% having attained at least a tertiary education. Participants reported having held their full driver’s licence for an average of 10.5 years, travelling 316.3 km per week and 96% identified as experienced drivers. Over half (52%) had used a stimulant at least once. More specifically, 36% acknowledged using amphetamines/speed, 40% had used MDMA/ecstasy and 44% had tried cocaine. One participant reported monthly use of amphetamines, while all others who reported either amphetamines/speed, MDMA/ecstasy and/or cocaine use, reported infrequent use, less than once a month. All participants indicated previous alcohol consumption, 88% had used cannabis and one (4%) had used an inhalant.

### Subjective measures

Participants reported no significant differences in perceived drug effects, including ‘strong’, ‘good’, ‘bad’ or ‘like’, between conditions (all *p* > 0.05). Prior to the drive, there was no significant difference in KSS scores between the placebo (4.96 ± 1.60) and methylphenidate conditions (4.92 ± 1.72) (*p* = 0.934), indicating similar levels of alertness in both conditions. Post-drive analyses revealed a significant increase in sleepiness within the placebo condition, with KSS scores rising to (6.56 ± 1.92) (mean difference = 1.60, t_24_ = 4.33, *p* < 0.001). In contrast, there was no significant change in the KSS scores pre- to post-drive in the methylphenidate condition (*p* = 0.408).

### Driving performance

Table 2 presents a summary of driving performance data, including means and standard deviations. For brevity, this section focuses on significant main effects and interactions. Linear fixed-effects model analyses revealed a significant treatment effect for SDLP (cm). Notably, SDLP was reduced, indicating less weaving following methylphenidate administration compared to placebo (mean difference = −1.33, *p* = 0.001, 95% CI = −0.52 to −2.14). Further analysis demonstrated that relative to placebo, SDLP significantly decreased with methylphenidate at both 30 (mean difference = −1.54, *p* = .029, 95% CI = −0.17 to −2.91) and 40 min (mean difference = −2.87, *p* = 0.003, 95% CI = −1.12 to −4.62), but not at 10 or 20 min (both *p* > 0.05, see Figure 2). A significant treatment-by-time interaction was observed for SDS (km/hr) (F_3, 165.08_ = 3.11, *p* = 0.028), whereby a treatment effect emerged at 40 min but not at 10, 20 or 30 min. At 40 min, SDS decreased under methylphenidate relative to placebo (mean difference = −0.56, *p* = 0.014, 95% CI = −0.10 to −0.123), signifying improved speed maintenance.

**Table 2.** Driving performance overall and across time (10, 20, 30 and 40 min) and treatments (placebo and methylphenidate) presented as mean (± standard deviation).

Measure	Time	Placebo (*n* = 25)	Methylphenidate (*n* = 25)
SDLP (cm)	Overall	23.61 (4.64)	22.24 (4.24)**
	10	23.06 (4.21)	22.62 (3.50)^a^
	20	23.37 (5.01)	22.61 (5.24)
	30	23.40 (3.83)	21.86 (3.84)*
	40	24.63 (5.45)	21.87 (4.35)^b^**
SDS (km/h)	Overall	1.80 (0.92)	1.65 (0.78)
	10	1.90 (0.53)	1.97 (0.84)^a^
	20	1.56 (0.86)	1.60 (0.73)
	30	1.69 (0.84)	1.56 (0.65)
	40	2.06 (1.28)	1.47 (0.85)^b^*
SV (wheel position)	Overall	0.0017 (0.0011)	0.0016 (0.0009)*
	10	0.0021 (0.0011)	0.0020 (0.0009)^a^
	20	0.0015 (0.0009)	0.0015 (0.0007)
	30	0.0014 (0.0011)	0.0012 (0.0008)
	40	0.0018 (0.0011)	0.0017 (0.0010)^b^

Asterisks indicate significant differences from placebo (*p < 0.05; **p < 0.01, Bonferroni-corrected).

Data missing for ^a^*n* = 1 and ^b^*n* = 2 due to equipment failure.

**Figure 3.**
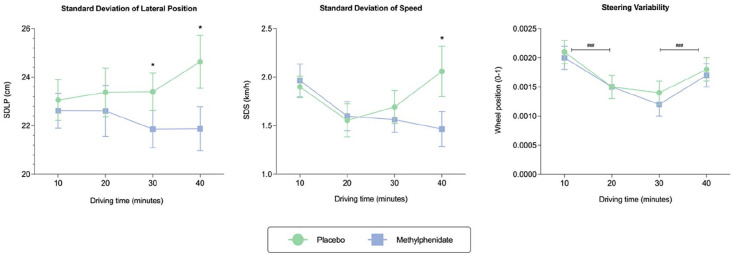
Mean values for standard deviation of lateral position (SDLP), standard deviation of speed (SDS) and steering variability (SV) across treatments (placebo and methylphenidate) at varying time points (10, 20, 30 and 40 min). Error bars represent ± standard error of the mean (SEM). The asterisk (*) symbol indicates statistical significance between conditions, with *p = 0.05. The hash symbol (^#^) denotes differences across time points, with ^###^*p* = 0.001.

A significant main effect of treatment was also observed for SV. Notably, there was a marked reduction in steering wheel movements when methylphenidate was administered compared to placebo (mean difference = −0.0001, *p* = 0.037, 95% CI = −0.0003 to −0.00001). A main effect of time for SV was also detected (F_3, 165.06_ = 18.71, *p* < 0.001). Post hoc analysis revealed a significant decrease in SV from 10 to 20 min (mean difference = −0.0005, *p* < 0.001, 95% CI = −0.0003 to −0.0008). Conversely, a significant increase in SV was observed from 30 to 40 min (mean difference = 0.0004, *p* < 0.001, 95% CI = 0.0001–0.0006) (Figure 3).

### Ocular parameters

Summary data including means and standard deviations for ocular outcomes are presented in Table 3.

**Table 3.** Ocular outcomes overall and across time (10, 20, 30 and 40 min) and treatments (placebo and methylphenidate) presented as mean (±standard deviation).

Measure	Time	Placebo (*n* = 19)	Methylphenidate (*n* = 19)
SGE (bits)	Overall	0.29 (0.19)	0.33 (0.18)
	10	0.26 (0.20)	0.32 (0.22)
	20	0.28 (0.18)	0.34 (0.19)
	30	0.34 (0.21)	0.33 (0.19)
	40	0.30 (0.16)	0.33 (0.14)^a^
GTE (bits)	Overall	0.43 (0.22)	0.46 (0.21)
	10	0.43 (0.24)	0.51 (0.21)
	20	0.43 (0.20)	0.44 (0.20)
	30	0.40 (0.23)	0.48 (0.24)
	40	0.45 (0.21)	0.41 (0.16)^a^
Fixation duration (ms)	Overall	397.66 (87.87)	383.39 (108.31)*
	10	407.21 (90.45)	399.39 (130.96)
	20	400.87 (94.35)	382.48 (104.95)
	30	399.41 (88.07)	379.22 (98.88)
	40	383.15 (82.90)	372.00 (100.47)^a^
Fixation rate (count/min)	Overall	79.84 (19.05)	84.35 (14.41)
	10	78.11 (22.39)	83.14 (12.84)
	20	79.22 (20.16)	82.74 (15.73)
	30	80.31 (18.97)	85.52 (14.60)
	40	81.78 (14.79)	85.98 (15.08)^a^

Asterisks indicate significant differences from placebo (*p < 0.05, Bonferroni-corrected).

Data missing for ^a^*n* = 1due to equipment failure.

There was a significant treatment effect for fixation duration (ms), with methylphenidate reducing fixation duration compared to placebo (mean difference = −12.68, *p* = .029, 95% CI = −1.30 to −24.057). There was also a significant main effect of time for fixation duration (F_3, 142.94_ = 4.29, *p* = 0.006). Post hoc analysis revealed that fixation duration was significantly reduced from 10 to 40 min (mean difference = −27.19, *p* = 0.003, 95% CI = −6.83 to −47.55). There was no main effect of treatment or time, or their interaction for SGE, GTE or fixation rate (all *p* > 0.05).

### Perceived driving quality and simulator sickness

Participants self-reported improved driving performance under the influence of methylphenidate relative to placebo (*t*_23_ = 3.81, *p* < 0.001). Specifically, participants rated their driving as better than normal following methylphenidate administration (12.71 ± 4.28), and worse than normal following placebo (9.36 ± 3.84). Additionally, participants experienced mild simulator sickness in both the placebo (4.12 ± 2.73) and methylphenidate conditions (3.46 ± 3.09), with elevations in fatigue, eye strain, difficulties focusing and concentrating being reported. No significant differences were observed in the overall incidence or intensity of individual simulator sickness symptoms between conditions following the driving task (all *p* > 0.05).

## Discussion

This study investigated the acute effects of 10 mg methylphenidate on driving performance and concurrent ocular activity in healthy adults. Methylphenidate administration resulted in significant improvements in vehicle control, evidenced by a decrease in SDLP and SDS compared to placebo, with the effects most pronounced between 20 and 40 min of driving. Furthermore, we found a significant treatment-by-time interaction for SDS, with methylphenidate improving the capacity to maintain a steady speed between 30 and 40 min of the driving task, which was absent at earlier intervals. These improvements in driving performance under methylphenidate were subjectively acknowledged by participants, who reported a higher perceived driving compared to placebo. This subjective assessment is consistent with the objective data, demonstrating that methylphenidate not only enhances driving abilities but also maintains driver attention throughout the task. Additionally, participants reported a significant increase in sleepiness from the start to the completion of the driving test in the placebo condition, which was not present under methylphenidate. This absence of increased sleepiness coupled with improved performance suggests that methylphenidate may protect against typical performance decrements associated with prolonged task engagement and driving time (Verster and Roth, 2013).

Regarding gaze behaviour, methylphenidate administration significantly reduced the duration of fixations compared to placebo. While longer fixation durations are typically associated with increased cognitive effort required to process visual stimuli (Marquart et al., 2015), shorter fixations might indicate more efficient visual information processing. This efficiency potentially aids in improving the driver’s ability to process basic driving cues, such as recognising traffic signs and maintaining an appropriate following distance, as evidenced by the improved driving performance discussed above. These findings align with the known effects of methylphenidate to enhance monoamine activity within the prefrontal cortex and basal ganglia, both crucial in controlling oculomotor action and goal-oriented behaviour (Milea et al., 2007; Volkow, 2002).

Despite these positive effects on fixation duration, the improvements did not extend to overall visual scanning efficiency, as there were no significant effects for GTE or SGE. Visual scanning efficiency involves the optimal mediation of bottom-up (i.e. visual complexity in the environment) and top-down (e.g. prior knowledge and task demand) influences on gaze control (Shiferaw et al., 2019a). Unlike methylphenidate, which subtly increases dopamine levels by inhibiting reuptake and thereby enhances focus and sustained attention useful for driving, more potent psychostimulants like methamphetamine and MDMA induce a significant dopamine release (Ares-Santos et al., 2013), depending on the route of administration. In contrast to oral tablets, methamphetamine is more commonly smoked or injected, leading to a more rapid and intense dopaminergic response. This excessive dopaminergic activity can overwhelm neural pathways, leading to scattered attention and disrupting the processing of multiple stimuli simultaneously. This differential modulation of the dopaminergic system by these drugs may account for their varied impacts on visual scanning abilities, highlighting the complexity of how stimulants affect cognitive functions in different contexts (Hayley et al., 2021).

While the use of technology to monitor gaze behaviour offers considerable potential for identifying real-time impairment and thereby reducing the incidence of road trauma, the subtle changes observed in our study, particularly the isolated reduction in fixation duration, provide only limited support for enhancing the capabilities of DMS. The absence of adverse effects on driving performance that might indicate an increased risk of collision, coupled with the general lack of strong ocular evidence of impairment, emphasise the reliability and potential precision of such monitoring technologies. Our findings contribute to a deeper understanding of how pharmacological distinctions between prescribed (e.g. methylphenidate) and illicit stimulants (e.g. methamphetamine) are reflected in discernible driving behaviours. This research builds on prior studies that have explored specific amphetamine-induced alterations in gaze behaviour while driving (Hayley et al., 2024), suggesting that while some stimulants may enhance certain cognitive functions beneficial for driving, others may lead to detrimental effects. By further delineating these pharmacological impacts, we can better tailor DMS technologies to detect and respond to various degrees of drug-induced driving impairments, potentially integrating more nuanced behavioural markers beyond basic gaze metrics to improve road safety outcomes.

### Limitations and future research

Given the relatively low complexity of the simulated highway drive, the demands on gaze behaviour, the necessity for frequent and varied eye movements may be low. Consequently, the repetitive nature of the task may have resulted in a ceiling effect, in which the range of required eye movements is inherently limited, regardless of the internal state of the driver. The outcomes of this study should be contextualised with the recognition that real-world driving involves navigating through a broad spectrum of environments. In naturalistic and more challenging driving scenarios that involve direct safety risk thus demand greater visuospatial attention, such as manoeuvring through urban roads or navigating heavy traffic, a more significant effect of methylphenidate on gaze behaviour and scanning efficiency might become apparent. Furthermore, we used a relatively low, acute dose of methylphenidate in this study, which reflects typical introductory therapeutic doses. Thus, we acknowledge that this may not fully capture alterations associated with higher acute doses or patterns of change following chronic usage, which are arguably more common in real-world misuse scenarios and likely associated with RTCs. The dose-related limitation in the present study is reflective of much of the clinical experimental work assessing acute amphetamine effects, which is limited by complex ethical and methodological constraints. Future research should therefore consider varying the dosing regimens, including higher and repeated doses over extended periods, and incorporating more complex driving simulations to better replicate real-world conditions, and assess the interaction of methylphenidate intoxication with unexpected or rapidly changing road environments. Additionally, incorporating direct markers of cortical and subcortical processing to complement gaze metrics would provide a more comprehensive understanding of the underlying neural mechanisms driving behaviour.

## Conclusion

This study demonstrated that an acute 10 mg dose of methylphenidate demonstrated protective effects against performance degradation commonly observed during prolonged, monotonous driving, evidenced by improvements in vehicle control and speed maintenance relative to placebo. The limited changes in broader ocular metrics such as GTE and SGE suggest that while methylphenidate enhances specific aspects of cognitive function, it does not universally improve visual scanning efficiency. There is a clear need for further research in this area, particularly studies aimed at identifying more pronounced alterations in ocular behaviour caused by methylphenidate and other psychostimulants. Considering the broad range of effects elicited by this class of drugs, it is important for research to clearly outline the spectrum of these effects such that thresholds can be established to enable detection of impairment by monitoring technologies and reduce safety risk. Without evidence from scientific investigations safety technologies such as DMS cannot be adequately equipped with capabilities to protect road users against the safety risk misuse of these drugs present. Additionally, studying these effects under various driving conditions would provide more comprehensive insights, better guiding the development of next-generation DMS technology.
